# Cardiovascular Responses Associated with Daily Walking in Subacute Stroke

**DOI:** 10.1155/2013/612458

**Published:** 2013-02-14

**Authors:** Sanjay K. Prajapati, Avril Mansfield, William H. Gage, Dina Brooks, William E. McIlroy

**Affiliations:** ^1^Graduate Department of Rehabilitation Science, University of Toronto, 500 University Avenue, Toronto, ON, Canada M5G 1V7; ^2^Toronto Rehabilitation Institute, UHN, 550 University Avenue, Toronto, ON, Canada M5G 2A2; ^3^Heart & Stroke Foundation Centre for Stroke Recovery, Sunnybrook Health Sciences Centre, 2075 Bayview Avenue, Toronto, ON, Canada M4N 3M5; ^4^Department of Physical Therapy, University of Toronto, 500 University Avenue, Toronto, ON, Canada M5G 1V7; ^5^Faculty of Health, School of Kinesiology & Health Science, York University, 4700 Keele St., Toronto, ON, Canada M3J 1P3; ^6^Department of Kinesiology, Faculty of Applied Health Sciences, University of Waterloo, 200 University Avenue West, Waterloo, ON, Canada N2L 3G1

## Abstract

Despite the importance of regaining independent ambulation after stroke, the amount of daily walking completed during in-patient rehabilitation is low. The purpose of this study is to determine if (1) walking-related heart rate responses reached the minimum intensity necessary for therapeutic aerobic exercise (40%–60% heart rate reserve) or (2) heart rate responses during bouts of walking revealed excessive workload that may limit walking (>80% heart rate reserve). Eight individuals with subacute stroke attending in-patient rehabilitation were recruited. Participants wore heart rate monitors and accelerometers during a typical rehabilitation day. Walking-related changes in heart rate and walking bout duration were determined. Patients did not meet the minimum cumulative requirements of walking intensity (>40% heart rate reserve) and duration (>10 minutes continuously) necessary for cardiorespiratory benefit. Only one patient exceeded 80% heart rate reserve. The absence of significant increases in heart rate associated with walking reveals that patients chose to walk at speeds well below a level that has meaningful cardiorespiratory health benefits. Additionally, cardiorespiratory workload is unlikely to limit participation in walking. Measurement of heart rate and walking during in-patient rehabilitation may be a useful approach to encourage patients to increase the overall physical activity and to help facilitate recovery.

## 1. Background

Regaining independent ambulation is important to those with stroke [1, 2] and is the most frequently reported rehabilitation goal [3, 4]. Therefore, walking should be an integral part of in-patient rehabilitation. However, accelerometer-based monitoring of walking activity has revealed that the amount of daily walking completed by individuals with stroke during in-patient rehabilitation is low [5, 6]. Importantly, the majority of walking bouts are of short duration (<1 minute) [5–7] and typically involve walking to essential activities (e.g., washroom, dining area, or therapy) [5]. 

While activity monitors provide insight into total daily activity [5–10], they do not inform the possible determinants or consequences of this activity. Aerobic capacity is reduced in the early months following stroke [11–13]. Furthermore, poststroke gait is inefficient, and there are increased aerobic demands on those with stroke when walking compared to healthy controls, even when walking at the same speed [14]. Therefore, individuals with stroke are closer to their maximal aerobic threshold when walking than healthy controls. This potentially limits the intensity (i.e., speed) and total duration of walking activity during daily life.

Walking can be a valuable means to improve aerobic capacity [15]. Aerobic exercise can help to improve aerobic capacity following stroke and improve recovery from stroke [16, 17]. Furthermore, there is evidence that increased amount of rehabilitation early after stroke improves recovery [18, 19]. However, limited resources within rehabilitation hospitals may impede the ability to provide formalized or structured aerobic training. Unstructured and unsupervised activities, such as daily walking, provide an opportunity to benefit aerobic fitness afterstroke. Presently, it is not known if in-patients engage in episodes of walking that have aerobic benefit outside of therapy.

This study aims to answer two questions: (1) does unsupervised, unstructured daily walking activity provide aerobic benefit to individuals after stroke and (2) is daily walking activity limited after stroke due to increased energy demands of walking? This initial study was specifically focused on a sample of stroke patients who were able to walk independently and who resided within a rehabilitation hospital. We view this subacute phase to be particularly important for enhancing aerobic training after stroke. To address the first objective, we determined if individuals with stroke engaged in walking bouts that were at least 10 minutes long at an intensity of 40%–80% of heart rate reserve (HRR), totaling at least 20 minutes per day [15]. To address the second question, we determined the duration of walking activity that reached or exceeded the aerobic threshold of 80% of HRR [20]. The latter, if it occurred, would reflect that the challenge of everyday walking may pose a potential barrier to being more active. 

## 2. Methods

### 2.1. Patients

We included individuals who were attending in-patient rehabilitation following stroke and who were able to walk independently without supervision (with or without use of a walking aid). We excluded patients who used heart-rate (HR) altering medication (e.g., beta-blockers) as HR response would be variable depending on when medication was taken. Eight individuals volunteered to participate and provided informed consent. The study was approved by the institution's research ethics board, and study procedures were in accordance with institutional guidelines. Characteristics of the eight volunteers are presented in Table 1. Participants underwent clinical assessment of gait, functional balance, and motor impairment. Spatiotemporal characteristics of gait were collected using a pressure-sensitive mat (GAITRite, CIR Systems Inc., Havertown, PA, USA). Participants walked across the mat three times at their preferred speed and the location and timing of each footstep were sampled at 30 Hz. We then calculated walking speed, cadence, and temporal symmetry [21]. Motor impairment was assessed using the Chedoke-McMaster Stroke Assessment (CMSA) [22]. Functional balance was assessed using the Berg Balance Scale (BBS) [23].

### 2.2. Ambulatory and Heart Rate Data Acquisition

**Figure 1:**
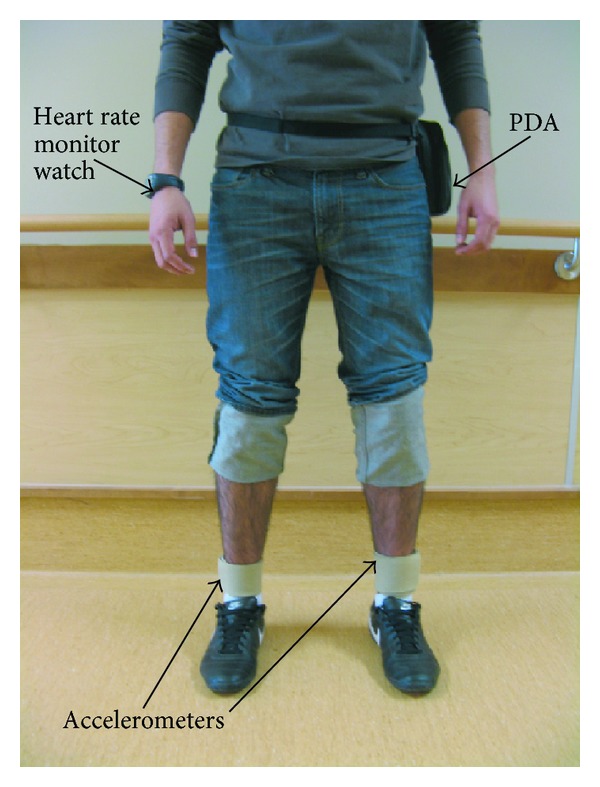
Placement of the ABLE system on a patient. Highlighted in the figure is the personal digital assistant (PDA) data logger worn around the waist of the patient, bilateral placement of accelerometer straps worn superior to the lateral malleolus, and heart rate wrist watch data logger.

The ABLE system [5] (Figure 1) was used to collect ambulatory data. The ABLE system is comprised of two triaxial accelerometers (SparkFun Electronics, Boulder, CO, USA) worn bilaterally around the ankles, which transmit data wirelessly to a personal digital assistant (PDA) (Hewlett Packard, Palo Alto, CA, USA) worn around the waist. The accelerometers were placed just proximal to the lateral malleoli using custom ankle sleeves, and the PDA was secured to the participant's waist using a polyester belt and pouch. Data from each accelerometer unit were recorded on the PDA at 50 Hz. HR data were acquired using a commercially available HR monitoring system (Polar Electro, Kempele, Finland). Participants wore a chest strap and a wristwatch. Heart beats were recorded by the chest strap and transmitted wirelessly to the wristwatch, which logged HR data at 0.2 Hz. The two collection systems were synchronized by initiating data collection in tandem. 

Patients were fitted with the ABLE and HR monitoring systems in the morning after routine activities were completed (e.g., bathing). The investigator checked every one to two hours to ensure that there was no discomfort and that all devices remained operational. Data collection continued for approximately eight hours, between approximately 9 am and 5 pm.

Periods of walking activity were identified and delimited to “bouts” of walking in our analysis. A bout of walking consisted of at least 10 consecutive steps; shorter bouts would not likely have yielded a measurable HR response. Individual bouts of walking were differentiated by a pause of at least 5 seconds prior to the next bout of walking [5]. 

### 2.3. Physiological Change Detection

The Karvonen formula [20] was used to determine the cardiovascular intensity (i.e., % of HRR) during bouts of walking. Using HR collected from the HR monitor (HR_observed_), the % of HRR was determined for each bout of walking by using the following modified Karvonen formula:

(1) $$\begin{array}{c} \%\text{HRR}\;=\frac{\left({\text{HR}}_{\text{observed}}-{\text{HR}}_{\text{rest}}\right)*100}{\left({\text{HR}}_{\max}-{\text{HR}}_{\text{rest}}\right)}. \end{array}$$

HR_max⁡_ was the estimated maximum HR and was determined by subtracting the participant's age from 220 [15]. While the patient remained seated, resting HR (HR_rest_) was determined by recording the lowest HR measured within the initial 10-minute period of collection. For each identified bout of walking, HR response (HR_observed_) for that specific bout was determined by averaging the three highest consecutive HR measures. This approach was taken in order to acquire a sustained HR response over 15 s (e.g., three HR measurement points), as opposed to using a single-point HR, which has the potential to be influenced by transient mutant HR responses. 

## 3. Results

Mean data collection duration was 8.4 hours (standard deviation: 0.8 hours). Six of the eight participants required a walking aid during both daily walking and clinical data collection (Table 1). At the time of collection, participant B had begun independently ambulating only recently with a walking aid after having been limited to a wheelchair since the onset of her stroke. 

### 3.1. Characteristics of Daily Walking

Walking characteristics are outlined in Table 2. The mean number and duration of bouts throughout the collection period were 62.6 bouts (standard deviation: 21.4 bouts) and 57.1 s (standard deviation: 31.6 s), respectively. Overall, 80.8% of all walking bouts were less than 1 min in duration, only 1.8% of all bouts were greater than 5 minutes, and only two walking bouts were greater than 10 minutes. Average step count was 3,708 steps (standard deviation: 1,452). Participant B demonstrated the lowest number of walking bouts with 33 (1,774 steps), while participant E demonstrated the greatest number of walking bouts with 91 (4,778 steps). The single longest bout duration was 13 minutes by participant F, which was performed during structured therapy.

### 3.2. Did Patients Meet Recommended Physiological Intensities and Durations for Aerobic Exercise during Daily Walking? 

**Figure 2:**
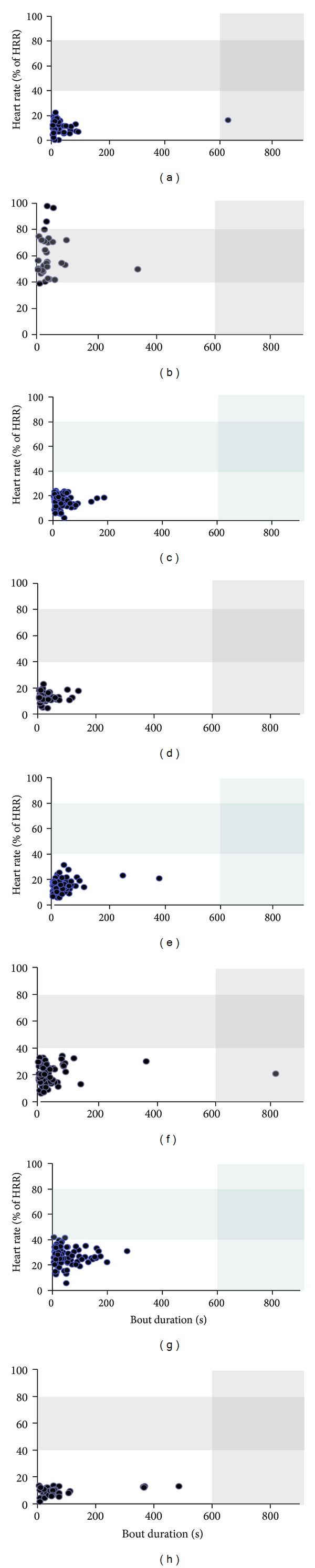
Mean heart rate response, expressed as a percentage of heart rate reserve (HRR), versus duration of walking bout throughout the collection period for all participants. Each point represents a single bout of walking performed during the collection period. The shaded areas indicate the recommended intensity (40%–80% maximum heart rate) and duration (10 minutes of continuous walking) of walking.

Overall, none of the participants fulfilled both requirements of duration (bouts ≥10 minutes long for a total of 20 minutes per day) and intensity (% of HRR ≥ 40%) for aerobic benefit (Figure 2). This was most profoundly limited by the duration of the bouts of walking as detailed in the preceding section. With respect to amplitude only 3.1% of all bouts detected, occurring in only two participants, were found to be above 40% HRR. The overall average intensity for bouts of walking was 19.4% HRR. Of the 8 participants tested, only participants B and G exhibited bouts of walking that exceeded the minimum 40% HRR. Participant B had the greatest number of bouts over 40% HRR, which occurred in 31 bouts (93% of their total bouts); participant G exhibited 3 bouts (3.3% of their total bouts) during which HRR exceeded 40%. The remaining participants did not present walking intensities above 40% HRR in any bout. 

### 3.3. Did Patients Exceed Recommended Intensities during Daily Walking? 

Participant B was the only participant found to exceed the 80% HRR threshold for any bout of walking (Figure 2). This participant presented 3 bouts (9% of her total bouts) above 80% HRR which ranged from 85.8% to 97.5% HRR. These bouts were not long in duration (<1 minute) and were not performed at high cadences (70–74 steps/minute). These bouts were performed while the participant walked with a rollator on a pedestrian pathway outside the hospital under the supervision of a physiotherapist. 

## 4. Discussion

The present investigation sought to determine the extent to which individuals with stroke met or exceeded the recommended cardiovascular intensities of everyday walking activity during in-patient rehabilitation. Together, the quantity and intensity of everyday walking have the potential to positively or negatively influence poststroke recovery. Such information provides insight into the potential value of everyday walking and contributes to the understanding of adaptations to current rehabilitative practices that can be made to maximize time spent in therapeutically beneficial activities. 

### 4.1. Participants Did Not Meet Recommended Duration and Intensity of Walking Activity

In agreement with previous work [5, 6, 24] the total amount of spontaneous walking activity was low. As our prior study has also indicated [5], durations of walking throughout an in-patient's day primarily consist of short bouts (e.g., less than one minute). Although there were no data available on healthy individuals for comparison, it is possible that short durations of walking activity observed are not just specific to patients with stroke, but more broadly reflect common walking patterns for inside environments. However, among all eight participants, only one spontaneous walking bout was longer than 10 minutes in duration; one other walking bout was longer than 10 minutes, but this occurred during scheduled physiotherapy. In terms of step counts, it is recommended that individuals with disability take at least 6,000 steps per day, of which 3,000 should be engaged in moderate-to-vigorous physical activity [25]. No participant in the current study attained 6,000 steps in one day. 

The majority of walking was at <40% HRR, and no participant completed 3,000 steps at a moderate-vigorous level. The overall mean of 19.4% HRR for everyday walking (including periods of structured therapy) was similar to the 24.2% (SD 21.2) HRR of walking found in patients with stroke performing standing and walking tasks exclusively during physical therapy [26]. HRR provides a more informative measure of intensity than cadence or walking speed as it is linked to the patients' aerobic capacity. While the present and previous studies were based on single day “snapshot” of activity, the low duration and intensity of activity for in-patients residing in a rehabilitation facility are striking. The absence of therapeutically beneficial walking activity may be attributed to factors such as short durations, low walking speeds, and the purpose for which patients walked. It is likely that low walking duration and low intensity levels may well be associated with more conventional walking activities, such as activities of everyday living (e.g., going to meals). Subsequent measurement will need to be extended to longer periods of time. However, the modest HR responses confirmed other work that monitored HR through the day [27] leading to the view that such moderate within-day HR responses may be “typical” of patients' experiences. 

### 4.2. Excessive Walking-Related Cardiovascular Load Does Not Limit Walking Duration

We sought to determine if HR response might be a limiter to walking duration and a potential barrier to activity. As noted the occurrence of HR response greater than 80% HRR was rare and occurred in only one participant (B). This participant had only begun to start walking independently one day prior to data collection after having been mostly wheelchair bound for one month following her stroke. Therefore, this individual was likely extremely deconditioned as a result of limited mobility and, consequently, reached the threshold of her aerobic capacity easily during daily walking. Limited aerobic capacity potentially limited total walking time for this participant; she had the lowest total walking duration and fewest total steps among all participants. However, reduced balance control and increased motor impairment may also have limited total daily walking. Participant B also had the lowest BBS score, CMSA scores, and self-selected walking speed. Increased motor impairment may have increased the energy demands of walking and caused participant B to reach the threshold of aerobic capacity more easily than other participants [14, 28]. Alternatively, reduced walking duration may have been a strategy to prevent a fall given impaired balance control [24]. 

Among the remaining seven participants, there was no evidence that reduced aerobic capacity limited total daily walking activity. No other participant attained >80% HRR while walking during the day. Furthermore, long-duration walking bouts (e.g., 5 minutes or longer) were not associated with increased HR response. Therefore, aerobic capacity did not limit frequency of long-duration walking bouts. The barriers to increasing total spontaneous walking activity following stroke remain to be determined. While HR was typically low, it is possible that patients' perceived fatigue caused them to limit walking speed and time. We did not record perceived exertion or fatigue in the current study; this should be considered in future work. In hospital, patients are likely to be reluctant to walk outside, particularly if balance control is impaired or if the weather is poor. Reduced balance control may play a role, and patients may limit physical activity due to fear of falling [29, 30]. Long-duration walking bouts may be influenced by the size of the unit or the length of the corridors within the hospital, although the current unit features an approximately 50 m long hallway where patients can walk unencumbered. Such distances would provide a much greater opportunity for someone to walk indoors than would be possible in many indoor living settings in the community. However, patients who were transferred from in-patient care to the community were found to increase bout durations in the community, amounting to an extra 30 minutes of activity per day [6]. Such an increase may be partially attributed to opportunity and willingness to walk outside and participate in community activities [6] or may reflect the improved functional capacity at the time after discharge from in-patient rehabilitation. Finally, patients may not be aware of their limited walking activity, and interventions may be required to increase spontaneous walking activity during in-patient rehabilitation. Additional work is required to determine the factors contributing to reduced daily walking activity during in-patient stroke rehabilitation.

### 4.3. Clinical Significance

Profiling the relationship between ambulatory activity and HR response can have important health-related implications to poststroke rehabilitation. The absence of activity that may benefit cardiorespiratory health and fitness during supervised and unsupervised periods of the day highlights a larger problem associated with conventional in-patient care. Feasibility studies have demonstrated that structured equipment-based exercise programs can be implemented safely and without negative effects on conventional therapy [17]. Patients receiving aerobic training in addition to standard care have significant improvements in indices of neuromuscular control and functional ambulation [17]. However, these exercise programs require therapist supervision and other resources such as space and equipment. These results can be viewed as a missed or lost opportunity for supplementary rehabilitation practice. The benefit of measuring both walking and HR, as in the present study, is to help clinicians consider periods of unstructured activity. Emphasizing additional therapeutically relevant activities throughout the day may be one method to better address “down” time frequently experienced by patients [31, 32]. However, it can be argued that simply requesting patients to engage in additional walking activities outside of therapy may not occur or may occur at inadequate intensities or durations to provide meaningful improvements in cardiovascular health. By using heart rate and activity monitors to identify the absence of walking bouts required for therapeutic benefits, clinicians would be able to better guide treatment decisions regarding walking exercise programs. Future studies will need to examine intensity and duration of patient activity across multiple days to develop a better understanding of patient activity levels and barriers to increased activity. In addition, the potential use of bout duration and its associated physiological response as an outcome measure for clinical practice requires further study [6]. 

## 5. Summary/Conclusions

This preliminary investigation between walking activity and task-related HR responses provides insight into the physiological demands associated with daily walking on patients residing in a rehabilitation hospital. These results indicate that daily walking (performed indoors) likely does not provide cardiorespiratory benefit within this group. Consequently, to achieve aerobic benefits from daily walking patients should be encouraged to increase the quantity of walking, and additional emphasis needs to be placed on increasing the intensity of walking if possible. Among those patients for whom the recommended walking intensity is not yet possible aerobic training should be formally included into structured therapy or performed using equipment that poses no risk of falling (e.g., recumbent stepper). Ideally, encouraging adaptations to daily walking activity, to increase both duration and intensity, may help promote and facilitate recovery after stroke and reduce the risk of subsequent vascular events.

## Figures and Tables

**Table 1:** Demographic and clinical characteristics of patients.

Participant	Gender	Age (years)	Time after stroke (days)	CMSA		BBS (score)	Resting HR (beats/min)	Gait speed (m/s)	Cadence (steps/min)	Temporal symmetry (ratio)
				Leg	Foot					
A*	M	59	13	4	4	51	64	0.54	72.8	1.26
B*	F	31	28	3	4	32	72	0.28	67.2	1.15
C*	M	76	14	5	6	51	59	1.11	103.5	1.08
D	M	57	25	4	4	38	51	0.83	93.9	1.10
E*	M	38	68	5	5	41	64	1.10	100.6	0.98
F	M	54	46	5	5	49	55	0.86	98	1.03
G*	F	63	30	4	3	44	82	0.53	81.5	1.29
H*	F	47	32	3	4	33	51	0.31	60.0	1.20

Mean		53.1	32	4.1	4.4	42.3	62.3	0.70	88.2	1.15
Standard deviation		14.2	17.9	0.8	0.9	7.7	10.7	0.33	14.4	0.96

*Denotes use of an assistive device (e.g., single-point cane/rollator) throughout data collection.

BBS: Berg balance scale; CMSA: Chedoke-McMaster stroke assessment; F: female; HR: heart rate; M: male.

**Table 2:** Summary of walking measures for each patient collected throughout the day.

Participant	Total collection time (hours)	Total walking time (minutes)	Number of walking bouts	Mean bout duration (s)	Total step count	Mean cadence (steps/minute)
A	7.06	33.3	46	43.4	2643	73.2
B	8.85	24.6	33	57.6	1774	79.2
C	7.86	31.6	55	34.5	3743	85.8
D	8.65	31.7	79	31.8	2201	78.5
E	8.91	59.3	91	45.8	4778	87.2
F	7.87	58.4	60	131.3	5621	103.6
G	8.41	78.3	89	52.4	5377	78
H	9.69	74.7	48	60.2	3532	61.6

Mean	8.4	49	62.6	57.1	3708	80.8
Standard deviation	0.8	21.2	21.3	31.6	1452	12.1
